# Europe’s Care Regimes and the Role of Migrant Care Workers Within Them

**DOI:** 10.1007/s12062-012-9063-y

**Published:** 2012-05-01

**Authors:** Alice Anderson

**Affiliations:** European Union in a Global Order, University of Amsterdam, Amsterdam, North Holland the Netherlands

**Keywords:** Ageing, Europe, Migration

## Abstract

This paper is an examination of the recent restructuring and subsequent convergence of European long-term care models. This paper also aims to highlight the increased role of migrant care workers and the need for great social and governmental recognition for all care providers. The provision of long term care is complex, divided between state, market and family providers; the state alone could not and does not act as the sole provider of care (Banks 1998). The extent to which different sectors are relied upon is largely dependent on the ideology of the country's welfare state (Timonen and Doyle 2007).

“Europe’s future depends to a great extent on its capacity to tap the strong potential of the two fastest growing segments in its population: older people and immigrants” (European Commission 2011).

This paper aims to explore the relationship between long-term care regimes and the role of migrant care workers within the European context. ‘Long-term care’ (Ltc) or ‘elder care’ can be defined as the provision of informal, formal and unregulated care assistance to older persons by family members, public, private and non-for-profit care services and migrants. Care is provided through a complex network of formal (institutional care facilities), informal (usually provided by family) and unregulated (grey market, at-home care). Care is still often thought to be a private, family, primarily female concern. The growing need for carers is the result of not only an increased number of people reaching old age with dependency needs but also the transformation of the female role in society. Traditionally, it was presumed that care was a private responsibility, it was expected that a daughter or daughter-in-law would provide care to older infirm relatives, this is no longer the case with increased geographical separation of family members including increased female labour force participation (Osterle and Meichenitsch 2008). As Henk Nies from the INTERLINKS project stated with “100 million known carers across the EU, 19 million carers working for more than 20 h a week and 80 % of all care being provided by informal carers, care provision needs to be high on the EU agenda”.^1^ This paper explores to what extent the employment of migrant workers within both the formal and unregulated sectors of care provision is a way to meet the growing need for carers, manager the cost of care provision and help potential carers (women) to match work and care responsibilities.

Many people in care are not only living longer but also healthier lives; however, this is not the case for everyone. Nor, are the number of years with disability generally declining as longevity increases. The demand for care providers and the cost of care provision will increase as Europe’s baby boom population enters into retirement (over 65 years) and particularly into old age (over 80 years) resulting in growing demand for elderly long-term care provision. The number of people aged 60 years and over, in the EU, is now increasing by more than two million people every year. This is about twice as many as were entering this age bracket in 2007 (European Commission 2009). In 2008 the Eurostat study forecast that the proportion of the EU25 population aged 65 and over will grow from 17.07 % in 2007, to 20.68 % in 2020, to almost 30 % by 2040 (Osterle and Meichenitsch 2008). The fastest growing age cohort is aged 80 years and over (Eurostat 2010; Hoff et al. 2010) and this is the population group with the greatest dependency. In 2011 the OECD reported that 50 % of people in receipt of long-term care were over 80 years old. Therefore, it can be predicted that the demand for long-term care is going to increase exponentially in the coming years.^2^

A serious challenge for the EU and the Member States is that if the need for long-term elder care is not adequately addressed the financial cost of care services on the state will explode as the number of people in need of care increases and the number of traditional potential carers’ decreases. The OECD reports that among OECD countries the current average spending on long-term care is 1.5 % of GDP. It is estimated that expenditure on long-term care is “likely to double or more by 2050” (OECD 2011). Both formal (institutional care, home care services, medical services) and informal care (traditionally family) are very costly with regard to financial, time and emotional costs. Although at least 70 % of care is received at home, 62 % of government expenditure for care is on institutional care (OECD 2011). While family care is technically free it can result in an under employment of the working age population and the burden of some members of the work force having to provide care in conjunction with work. As the demand for formal and predominantly informal long-term care is increasing, the national and traditional supply of carers is neither affordable nor adequate.

## Why Do We Need Care? What is Carer Migration? And is it Necessary?

For several decades there has been a demographic shift towards an ageing population taking place across the European continent, albeit at varying speeds. The demographic shift is due to a decrease in fertility rates and increased longevity. The number of children being born has universally decreased across Europe and the birth rate is falling below replacement levels in some Member States such as Germany and Italy. In the European Commission 2010 Demography Report a slight improvement to fertility rates was reported but at 1.6 it is still far below the replacement rate of 2.1 and will, at best, slow the rate of population decline in the EU. In Italy, one of Europe’s oldest nations with one of the lowest fertility rates, the population is projected to decline from the current 57 million to 41 million by 2050 (European Commission 2011). This coupled with the positive fact that people are now living longer and often healthier lives has resulted in a uniquely large proportion of the population reaching old age.

Increased female involvement in the formal labour force coupled with changing family structures have put a strain on the ability of Member States to provide care for elderly who need assistance. Traditionally, one female member of the family would provide care. The continued idea that a family member and in particular a female one will provide care is in direct conflict with the Lisbon and 2020 Strategy targets of increased labour force participation. Additionally, since families are having fewer children and there is greater need for everyone to engage in the formal workforce the pool of potential carers has diminished or put another way this means that children are more likely to be needed as carers of their elderly parents. The major challenges now facing long-term care provision across Europe are related to the supply of carers, both formal and informal, and the high cost associated with long-term care service provision. In 2007 the EU average for the number of people aged 65 or over in receipt of institutional care was just over 3 %. Altogether, about 8 % of people aged 65 and over in the EU are receiving some formal long-term care at home - but this ranges from 25 % in Denmark and over 20 % in the Netherlands to 7 % in Ireland, only 1 % in Lithuania and even less in Poland (Huber et al. 2009).

‘Carer migration’ is defined as the movement of care workers or immigrants involved in the provision of care assistance to older people. Migrants are already filling the labour gaps and providing essential care services in many European Member States. In the United Kingdom and in Italy the proportion of migrants among the paid care work force is as high as 1 in 4 and 1 in 2 respectively (OECD 2011). Within the formal sector migrant workers make up a large proportion of available and employed carers. Similarly in an attempt to overcome the shortages and strain of long-term care many families are employing migrant workers within private homes to provide care. This is unregulated but often preferred as it is less expensive than formal care for the recipient and their family and it means that the care recipient can remain at home which has consistently been found to be the preferred place of care for the recipient.

### Demographic Shift

There are three drivers of population change: fertility rates, life expectancy and migration (European Commission 2010). Within the EU27 there are about 5 million births a year, which outnumbers deaths by only several hundred thousand according to the European Commission 2010 Demography Report. Net migration for the EU is over a million a year which makes migration the single greatest driver of population growth in the EU (European Commission 2010: 2). Demographic changes affect international migration in several ways: rapid population growth combined with economic difficulties at home is likely to push people to move in search of work, and a declining and ageing population pressures countries to accept migrants.

**Fig. 1:**
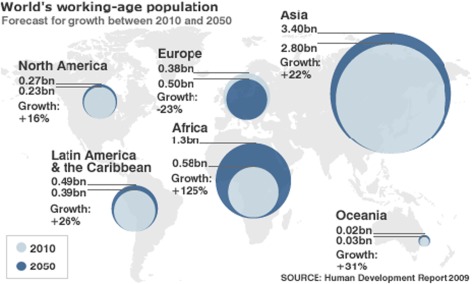
Working age population forecast for growth 2010–2050

Population ageing is a global issue effecting America, Japan, Korea and Australia but it is most pronounced within the European context. Figure 1 illustrates the growth predictions of the global working age population from 2010 to 2050. It clearly shows a North–south divide with younger, growing populations in the Southern hemisphere, much smaller growth predicted in the Northern hemisphere and a reduction of 23 % in Europe’s working age population by 2050.

### Formal and Informal Care in Ageing Societies

Long-term care is provided by both the formal and informal sectors. Informal care is usually provided by family or friends, while formal care has evolved to include state, private and non-for-profit agencies (Timonen and Doyle 2007). In general the formal care providers are paid and the informal unpaid but a range of arrangements exists including care allowances and long-term care insurance. The number of migrants working in the formal care sector has grown but to understand the extent of migrant care work the large numbers working in the grey market as unregulated carers in private homes also need to be taken into consideration (OECD 2011).

The need for long-term care and the responsibility for its provision have changed greatly over the last four decades. The demographic shift towards an ageing population has been accompanied by changes in the labour market and also in the family unit, so the role of women in society has dramatically changed. Traditionally care giving has been overwhelmingly unpaid women’s work, but it is increasingly recognised that care for the elderly is a larger societal concern. Demography and dependency ratios will play an important part in determining the sustainability of welfare states pensions.

Across Europe over the last 40 years more women have joined the labour force, birth rates have declined, (although a very slight increase was reported in 2010), and divorce has become more common. All these factors have contributed to a transformation of the traditional family. Social developments have changed the ability of women, in particular, to provide such extensive informal care (Döhner and Eickhoff 2008). The ‘male breadwinner’ model has been replaced by an ‘adult worker’ model which not only encourages but often requires the participation of both men and women in the formal labour market (Lutz and Palenga-Mollenbeck 2010: 425). This is reinforced in both the Lisbon and 2020 Strategies which aim to boost labour force activity. This shift has forced a re-organisation and re-distribution of care responsibilities within societies (Lutz and Palenga-Mollenbeck 2010). One outcome of the changing family model and role of women in society in the context of demographic ageing is that more elderly people are living alone. Within small families it is now more likely that an adult child will have to undertake some carer responsibilities but also that many older people are providing care to spouses. A recent study about family carers^3^ showed that there is still a strong desire to provide care to older relatives. However, eldercare is often a full-time job with little social or financial recognition and it has also been shown that people who care are more likely to need care themselves in later life (Döhner and Eickhoff 2008).

There has been a commodification of care; demand has been outsourced to the formal and paid migrant sectors and there has been a globalisation of care, as women migrate across the globe to cater for this growing market. As female labor force participation has increased demand for domestic workers has risen. Inadequate state provision and a move towards providing cash benefits for care services are intensifying this demand. Much of the new demand for care in private households is being met by non-EU nationals who are estimated to account for over 10 % of workers in this sector (Schwenken and Heimeshoff 2011) However, since much of this work is undocumented, its contribution to the European economy is likely to be much greater than reported. It was estimated that in 2008 about 100,000 female migrants were providing care to elderly people in Germany (Döhner and Eickhoff 2008). In 2010 it was reported that about 700,000 migrant care workers were employed in Italy; almost all of them by households in the unregulated sector (Interlinks 2010). Policy makers and the general public are only starting to become aware of the large number of older people in need of (affordable) care and the migrant carers who provide it (Döhner and Eickhoff 2008).

### A Convergence of European Care Systems

By looking at the past and present provision of care in some of the old European countries (Sweden, the Netherlands, France, Germany, the United Kingdom and Italy) it is possible to explore how different countries and their welfare models are responding to the demands of the demographic shift. The provision of long term care is complex, divided between state, market and family providers; nowhere can or does the state alone act as the sole provider of care (Banks 1998). The extent to which different sectors are relied upon is largely dependent on the ideology of the country’s welfare state (Timonen and Doyle 2007). Women’s working choices are, of course, strongly affected by the dominant care-provision ideology of their society (Lutz and Palenga-Mollenbeck 2010).

In Western Europe a spectrum of care provision exists; at one end lies the informal carer-led model, while at the other end the service-led model. The informal carer-led model is defined by limited governmental responsibility. In this model the state acts as a regulator rather than a provider of care; it is the users responsibility to find care, the government may then subsidise some of the financial cost. This model is heavily reliant upon family carers. At the other extreme, the service-led model aims to provide extensive service provision and to reduce the responsibility of the family. In this model direct financial support to users is more limited; care is provided through the use of extensive services, with the aim to make formal female employment and care compatible. Problems have arisen with both models; in the former the growing number of dependent older people living alone has increased the financial strain on the state and with more women in formal employment there has been a declining supply of informal carers. In the service-led model the high cost involved in providing extensive services and the demands for quality and flexibility of such services have put increasing strain on the capacity of the state.

In the 1990s countries such as Sweden and the Netherlands had relatively high levels of service provision and uptake, with high numbers of over 65s receiving the services provided. The UK had a medium degree of provision based predominantly on home care. Meanwhile Germany, France and especially Italy had fewer services and attempted to aid family support through a limited provision of cash programmes. Sweden had adopted the services-led model to the greatest extent with particular importance attached to home care services. Similarly the Netherlands provided a large number of services but also had a large investment in residential care. Both Italy and Germany encouraged more informal provision of care, although Germany did provide more residential care services. There has been a growing trend in the use and importance of cash benefits to support both dependent individuals and their family carers. Although little used in Sweden, where emphasis was on providing services, cash programmes had developed particularly in the UK, Germany and the Netherlands.

Regardless of the position of countries on the care provision spectrum 20 years ago, there has been a clear convergence among the new policies of these Member States. As financial constraints have increased with the growing numbers of dependent elderly, governments have begun to implement policies to support care at home; encouraging those who need care to look for in-house help. Such policies encourage the autonomy of the elderly, the caring capacities of their families and emphasise, at least in principle, greater freedom of choice. New policies have sought to combat the inefficiencies and limitations of previous cash programmes and service provision by rejecting the idea of two independent and mutually exclusive forms of care; in fact formal and informal care are now considered “complementary activities” (Pavolini and Ranci 2008: 250). This begs the question of where the migrant care workers fit in – particularly with regard to the eligibility for carers’ benefits. Public provision is seen as one element of care which aims to support informal care provision and increase the user’s freedom of choice (Pavolini and Ranci 2008).

Countries which began as predominantly informal care-led providers have increased public funding of services and support for informal carers. Meanwhile predominantly service-led countries have restructured their care services and concentrated the provision of care on those with higher dependencies while also increasing support for informal carers. Therefore both ends of the spectrum have taken steps to provide a more inclusive, effective and mixed provision of care (Pavolini and Ranci 2008).

It is important to note that this cannot be seen as a general overview of the care situation across the European Union and developments in other countries will reflect their own history, resources and values. Alber and Kohler (2004) underline the reliance upon family care in the new Member States but argue it is not merely due to a lack of public services but an indicator of the strength of family values in those countries.

Italy has made little effort to reform its system of care; there is still a heavy reliance on family responsibility either to provide care themselves or to outsource it to a private provider. The Italian system is so reliant on migrant carers that it could be argued that rather than being family dependent it is a ‘migrant dependent’ model (Pavolini and Ranci 2008: 257) Of course Italy is not the only country which is already heavily reliant on migrant care workers; in 1991 16 % of employed home care workers in Europe were foreign and this figure increased to 86 %, in 2005, the majority being employed as carers for the elderly (European Commission 2009). Clearly budgets are one factor limiting the development of services and supply but so too is the availability of human resources in the member states.

Increased demand for adequate long-term care services has put increasing pressure on governments to fund such services. In response governments are implementing policies which in theory aim to cater for the growing number of service users and to curb their growing financial strain on the state. Emphasis is often put on the fact that the new policies aim to support informal carers by providing benefits and cash programmes but it is not clear to what extent these cash-for-care programmes are naturally reaching family carers. The introduction of cash-for-care policies seek to establish a new balance between formal services and informal care, but also have implications for private funding of migrant carers in the unregulated market These new initiatives also recognise the major role of family care and consider how to support it rather than exploit it (Pavolini and Ranci 2008). It is often presumed that care work is an unskilled job but on the contrary it requires skills such as patience, empathy, emotional intelligence trust and high frustration management are all required and hard skills to learn (Lutz and Palenga-Mollenbeck 2010). Informal, family carers have been undervalued and unacknowledged in the care policy documents of many countries until relatively recently. Da Roit found, in her study “Caring Beyond Borders-Migrant Care Work in Europe”, that the higher the rate of care allowance and the looser the government regulations on payment the greater the number of migrants providing informal care.^4^

In Germany care insurance is highly regulated and paid to the care recipient. The payment aims to increase the recipient’s freedom of choice and sense of control. However, it can result in a lack of payment actually reaching informal care providers.

Timonen and McMenamin (2002) concluded that there were somewhere in the region of 100,000 people in Ireland providing informal care on a full-time or almost full-time basis and recent estimates are even higher. The vast majority, 80 %, of these carers were women. The disproportionate number of women providing informal care is likely to have detrimental long-term financial consequences by prolonging the discrimination of women in the labour force and reinforce the trap of women in low paid and undervalued employment (Glendinning et al. 2000). Even though the provision of cash-for-care payments can be perceived as a positive recognition of care work it can also trap carers, women, into undervalued physically and mentally demanding work.

This overview provides a look at the policies being implemented but not at how well they work in practice. Looking at the direction in which long-term care policies are moving, does not explore how, in reality, countries are meeting long-term care needs. Some policies that are put in place, while being sound in theory, may fail due to inflexibility or low carer and user take up. Many of the policies being put in place to cater for the growing older population are reliant upon a steady supply of available workers or family carers yet the question of how to maintain this supply is often not discussed.

### Supply and Demand

There is a need for governments to introduce policies which help workers maintain a work/life balance so as to encourage more people, specifically women, to enter the workforce and also to implement policies which curb the growing cost of formal care provision. Family care is not only the expressed preference for many elderly people it is also seen as a cost containing measure for the government (Timonen and McMenamin 2002).

**Fig. 2:**
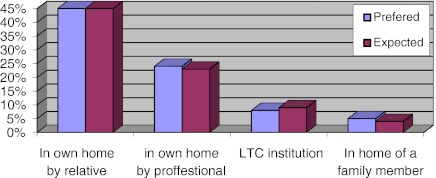
Expected and preferred way of getting assistance if one becomes dependent and needs regular help and long-term care % EU27: (Special Eurobarometer 2007: 95)

As Fig. 2 shows, expectations and preferences for different types of care provision are fairly evenly matched across the 27 EU Member States with a strong preference for home care. However, there are big differences between countries; the expectation to be looked after at home by a relative is very high in Eastern Europe; in Poland 70 % of respondents chose this option, while in Denmark only 20 % stated this as their preference (Eurobarometer 2007). Political discourses reinforce the cultural desire that care should be provided by the family at home (Lutz and Palenga-Mollenbeck 2010).

In reality the preference for home care often demands a much larger supply of carers than is available in that country and this leads to a heavy reliance on and use of the grey labour market. Grey labour market or unregulated work can be defined as involving people who do not have the legal right to employment in that country and in the case of the EU these are usually people from third countries (Döhner and Eickhoff 2008). It has been found that dependent persons would prefer to be cared for in their own home (Lutz and Palenga-Mollenbeck 2010). Various studies report this, such as the European DIALOG project, in which 14 different European countries participated (Höhn et al. 2008). When asked about help from informal, family carers (which could also include migrant care workers) 40–60 % stated that they would prefer that option, with the highest figure in Austria (77 %) and the lowest in Finland (39 %) (Höhn et al. 2008).

There is a heavy reliance on foreign labour in the care sector in Western European countries (Piperno 2010). Long-term care for the elderly is often provided by migrants coming from Eastern European countries (Döhner and Eickhoff 2008). Higher wages and employment opportunities encourage people to cross borders. Western Europe’s care services are sustained by workers from Eastern Europe (Döhner and Eickhoff 2008; Lutz and Palenga-Mollenbeck 2010). It can been observed that governments are content to turn a blind eye to the problem of unregulated and undeclared migrant workers providing long-term care as it is a short-term solution to the problem of increasing demand for care and limited finances (Lutz and Palenga-Mollenbeck 2010). Host governments and societies are benefiting from the cheap migrant labour that is providing essential services without having to provide many social services to the carers in turn. Tonken has argued that to prevent the exploitation and invisibility of migrant care workers the issue needs to go beyond the jurisdiction of individual Member States.^5^

By ignoring the situation of migrant care workers the government achieves two things;

- Cheap and flexible care provision;
- A continued societal misunderstanding about the important and positive role migrants play within societies.

A large number of people migrate into Europe either as carers (with and without qualifications) or they later become carers in both the formal and unregulated sectors. In a British study; ‘Migrant Care Workers in Ageing Societies’, it was found that in 2008 that 19 % of care workers and 35 % of nurses working in formal older care were migrants (Cangiano et al. 2009). By not properly addressing the role of migrant care workers governments are reinforcing the invisibility of this group within society. Carer migration is responding to the growing demand for workers aided by new migrant networks created through globalisation. Migrant flows are far from static and quickly respond to socio-economic needs; the rapid growth and transformation of countries means that their status as a sender or recipient of migrants can change quickly. Poland has been transformed since 2004 and is now experiencing both in and out flows of migrants simultaneously (Hoff et al. 2010).

Through the process of migration there is evidence of an extensive benefit available to host countries and to the immigrant workers themselves. In the host countries, the immigration of care workers is positively able to respond to the growing demand for care services. Yet the use of grey market and irregular employees endangers not only the quality of working life but also the quality and standards of these services (Piperno 2010). It is also unclear to what extent benefits are shared with the sender countries. It has been widely documented that remittances are an important source of income for migrant’s families and communities in the sender countries (Hoff et al. 2010). However, it would also appear that many sender countries are losing valuable and often skilled human capital at a time when they are needed in their home countries. Development in some third countries is continually defeated by the fact that after being educated and invested in by the taxpayers in their home country migrants leave to find higher wages and a better standard of living (Howse 2007). People are less willing to offer a care service (to a non-relative) in their own country if they can be paid more to do it in different location and this can lead to the issue of care drain (Piperno 2010).

## Conclusion

“Care is not a patriarchal concern for women, a type of secondary moral question or the work of the least well off in society. Care is a central concern for human life. It is time we begin to change our political and social institutions to reflect this truth.” (Tronto, 1993:180)

Europe’s demographic shift and the increasing demand for long-term care are becoming more evident and calling for urgent policy attention. However, the policy debate must be informed by a clear understanding of the subject. Care is provided through a complex mix of formal and informal carers and services. This paper has illustrated the need for clearer concepts of long-term care and for the categories of care providers. The growing demand for migrant care workers is a result of economic and social needs at both the formal and informal level. It is only possible to make relevant policy recommendations once the issues of formal/informal, contracted/unregulated and paid benefits in kind are clearly understood.

The growing numbers of migrant care workers cannot be understood outside of the social care system, policy regulations and culture of care within which they work. The role of migrant care workers within this web of care provision is a result of number of supply and demand factors. Care and care workers have gradually become a more prominent policy issue; care is no longer solely a private, family or female concern. On the demand side, the need for carers has increased as the numbers of old and dependent people has risen. In addition, the traditional supply of informal carers has been reduced through declining family sizes, more geographical mobility, as women have moved into the formal work force and are either unable or unwilling to provide long-term care to older relatives. Yet, at-home care remains the overwhelming long-term care preference for recipients and families. Migrant care workers have to been pivotal to both the formal and unregulated supply of care in Ireland; they have enabled a number of potential carers to enter the formal work force.

Similar to the complex nature of care provision, care is not a single policy issue but rather involves immigration, employment and health policies. At both the EU and national level immigration policies have been tightened in response to the recession and only skilled migration is being encouraged. Minimum income restrictions and work permit quotas have resulted in fewer qualified migrant nurses being employed below their skill level in the unregulated sector and the employment of migrant care workers has been pushed further into the grey market. And this is against a background of falling numbers in the working age population of the EU in the near future.

On a final note it has been found that the migration of care workers will be important to Europe’s economic recovery and demographic revival. However, this relationship can only be beneficial to all involved and in the long run when the policies in place promote an equitable and efficient relationship.

## Footnotes

1 Nies. H, 2011, Interlinks, Does Europe Care? Conference, Amsterdam.

2 Richelle. K, (DG of the EU Commission D-S of Employment, Social Affairs and Inclusion) 2011, Does Europe Care? Conference, Amsterdam.

3 EUROFAMCARE 2007

4 Da Roit. B, 2011, Does Europe Care? Conference, Amsterdam.

5 Tonken. E, 2011, Does Europe Care? Conference, Amsterdam.
